# Aspiration removal of orbitofrontal cortex disrupts cholinergic fibers of passage to anterior cingulate cortex in rhesus macaques

**DOI:** 10.1007/s00429-024-02776-6

**Published:** 2024-03-19

**Authors:** M. A. G. Eldridge, A. Mohanty, B. E. Hines, P. M. Kaskan, E. A. Murray

**Affiliations:** 1 Laboratory of Neuropsychology, National Institute of Mental Health, National Institutes of Health, Bethesda, MD USA; 2 Leo M. Davidoff Department of Neurological Surgery, Albert Einstein College of Medicine, Bronx, NY USA

**Keywords:** Rhesus macaque, Orbital Frontal Cortex, Anterior cingulate cortex, Acetylcholine, Cholinergic tracts, Emotional regulation, Cognitive flexibility

## Abstract

The study of anthropoid nonhuman primates has provided valuable insights into frontal cortex function in humans, as these primates share similar frontal anatomical subdivisions (Murray et al. 2011). Causal manipulation studies have been instrumental in advancing our understanding of this area. One puzzling finding is that macaques with bilateral aspiration removals of orbitofrontal cortex (OFC) are impaired on tests of cognitive flexibility and emotion regulation, whereas those with bilateral excitotoxic lesions of OFC are not (Rudebeck et al. 2013). This discrepancy is attributed to the inadvertent disruption of fibers of passage by aspiration lesions but not by excitotoxic lesions. Which fibers of passage are responsible for the impairments observed? One candidate is cholinergic fibers originating in the nucleus basalis magnocellularis (NBM) and passing nearby or through OFC on their way to other frontal cortex regions (Kitt et al. 1987). To investigate this possibility, we performed unilateral aspiration lesions of OFC in three macaques, and then compared cholinergic innervation of the anterior cingulate cortex (ACC) between hemispheres. Histological assessment revealed diminished cholinergic innervation in the ACC of hemispheres with OFC lesions relative to intact hemispheres. This finding indicates that aspiration lesions of the OFC disrupt cholinergic fibers of passage, and suggests the possibility that loss of cholinergic inputs to ACC contributes to the impairments in cognitive flexibility and emotion regulation observed after aspiration but not excitotoxic lesions of OFC.

**Supplementary Information:**

The online version contains supplementary material available at 10.1007/s00429-024-02776-6.

## Introduction

The orbitofrontal cortex (OFC) contributes to reward processing, value-based decision making, and affect, and its dysfunction is implicated in several neurological and psychiatric disorders. There is still much debate, however, over the range of functions supported by OFC, and around the identity of the specific mechanisms by which the OFC contributes to these behaviors (Bechara et al. 2000; Davidson et al. 2000; Rudebeck and Rich 2018).

The central dogma regarding the core function of OFC, supported by human neuropsychological literature, is that OFC exerts inhibitory control over emotional regulation and cognitive flexibility (Meyer and Bucci 2016; Mishkin 1964; Phillips 1999; Roberts and Wallis 2000). This idea is supported by data from individuals with OFC damage, in whom impairments in object reversal learning tasks–presumed to assess behavioral flexibility and inhibitory control–are correlated with disruption in emotional regulation (Rolls et al. 1994) and impulsivity (Berlin et al. 2004; Torregrossa et al. 2008). Similar effects are observed in macaques with OFC damage, including impairments in object reversal learning and Go/NoGo tasks (Jones and Mishkin 1972; McEnaney and Butter 1969; Rolls 2004; Izquierdo et al. 2004). More recent findings, however, have brought the dogma into doubt. We compared the performance of macaques with excitotoxic or aspiration lesions of OFC areas 11, 13, and 14 on three tasks: (1) object reversal learning to assess cognitive flexibility and inhibitory control; (2) an approach-avoid conflict task to assess emotional regulation; and (3) reinforcer devaluation to assess value-based decision making. Aspiration removals of OFC produced deficits on all three tasks (Izquierdo et al. 2004, 2005; Rudebeck et al. 2013). Excitotoxic lesions, which limit damage to local cell bodies and spare fibers of passage, produced no impairments on the tasks measuring inhibitory control and emotion regulation, but caused a deficit in value-based decision making of a magnitude comparable to that observed following aspiration lesions (Rudebeck et al. 2013). Importantly, in this within-laboratory comparison between effects of aspiration and excitotoxic lesions of OFC, groups of macaques were trained using the same behavioral procedures and test apparatus, and sustained lesions that were based on the same set of anatomical boundaries and were of similar extents. These observations led us to suggest that a revision of the inhibitory control hypothesis of OFC function may be in order. In addition, we proposed that fibers passing nearby or through OFC, which connect the frontal cortex with temporal cortex and subcortical regions, may be inadvertently damaged by aspiration lesions of OFC. Establishing the cause of the discrepancy between aspiration removals and excitotoxic lesions of OFC in macaques may shed light on the neural bases of these cognitive functions and the corresponding behaviors in humans.

Anatomical studies indicate that cholinergic fibers originating in the nucleus basalis magnocellularis (NBM) pass through or near the OFC on their way to other frontal cortical regions (Kitt et al. 1987; Lewis 1991; Selden et al. 1998). Thus, cholinergic fibers may be inadvertently compromised by aspiration removals of the OFC. One region that may be denuded of cholinergic innervation following OFC aspiration removal is the ventrolateral prefrontal cortex (VLPFC, area 12/47), a key node in cortical and subcortical networks implicated in inhibitory control (Aron and Poldrack 2005; Hiser and Koenigs 2018) and in rapid rule-based reversal in humans (Rolls et al. 2020) and in macaques (Chau et al. 2015). VLPFC is required for updating stimulus-value associations based on probability of reward in both macaques (Murray and Rudebeck 2018; Rudebeck et al. 2017; Folloni et al. 2021) and humans (Noonan et al. 2017). In addition, although aspiration lesions of VLPFC in macaques yield transient impairments in object reversal learning with deterministic outcomes (Iversen and Mishkin 1970), excitotoxic lesions of VLPFC yield no such impairment (Rudebeck et al. 2017), which, although far from conclusive, led us to consider the possibility that an alternative area supports this function. Another candidate region is the anterior cingulate cortex (ACC); this region supports inhibitory control and reversal learning in humans (Borst et al. 2014) and macaques (Chudasama et al. 2013; Crespo-García et al. 2022; Meunier et al. 1997), but is not essential for value-based decision making (Chudasama et al. 2013). Further, macaques with damage to sulcal ACC show altered responses in an approach-avoid task involving a fake snake (Rudebeck et al. 2006), which suggests an effect of ACC damage on emotional regulation. As such, loss of cholinergic innervation to the ACC, resulting in compromised function of this region, would be consistent with the behavioral effects of OFC aspiration lesions on inhibitory control and emotion regulation (Rudebeck et al. 2013).

As a first step in addressing the ‘fibers of passage’ hypothesis, the present study sought to assess whether cholinergic projections to ACC are compromised by aspiration removal of OFC. If the cholinergic fibers coursing nearby or through the OFC en route to ACC are damaged by aspiration lesions of OFC, we would expect to observe lower cholinergic innervation in hemispheres with OFC removal. To test this idea, we performed unilateral aspiration removals of the OFC in macaques and compared the density of cholinergic innervation within ACC, as measured by acetylcholinesterase (AChE) expression, in hemispheres with OFC aspiration lesions and intact hemispheres.

## Methods

### Subjects

Three adult male rhesus monkeys (*Macaca mulatta*: Monkey A, Monkey B, and Monkey C) served as subjects. All macaques were at least 4.5 years old, and their weights ranged from 5.1 to 10 kg. They were independently housed with access to water *ad libitum* and fed a diet of fruits, approved treats, and monkey biscuits twice daily. They were maintained on a 12-hour light-dark cycle.

### Apparatus and materials

#### Surgeries

Surgeries were performed in dedicated operating rooms under standard aseptic conditions (Izquierdo et al. 2004), and under veterinary supervision. Prior to the procedure, anesthesia was induced using ketamine hydrochloride (10 mg/kg, i.m.) combined with midazolam (0.1 mg/kg, i.m.), and the macaques were intubated. Anesthesia was maintained at a surgical level with isoflurane (1–4%, to effect) during the procedure. Macaques received an intravenous drip of isotonic fluids containing an antibiotic (Cefazolin), and vitals (body temperature, heart rate, SpO_2_, and expired CO_2_) were monitored throughout the surgery. The animal was first draped to establish an aseptic surgical field and a sagittal incision was made. The skull was exposed, bone flap removed, and *dura mater* reflected to provide access to the OFC. With the aid of an operating microscope, sulcal landmarks were identified on the orbital surface and the lesion boundaries were marked using electrocautery. Unilateral ablation of OFC was performed via subpial aspiration with a small-gauge suction tip using a combination of suction and electrocautery. The intended lesion was meant to include Walker’s areas 11, 13, and 14 (Walker 1940). The location and extent of the lesions were intended to replicate those described by (Baxter et al. 2000) and (Izquierdo et al. 2004). The anterior limit of the lesion was defined as an imaginary line between the rostral extents of the lateral and medial orbital sulci, and the posterior limit of the lesion was approximately 5 mm anterior to the frontal and temporal lobe junction. The medial limit of the lesion was the fundus of the rostral sulcus, and the lateral boundary was the fundus of the lateral orbital sulcus. Following the OFC removal, the dura was closed, the bone flap replaced, the incision was sutured in anatomical layers, and the animal was allowed to recover from anesthesia. Prior to discontinuing the gas anesthesia, macaques were administered ketoprofen (10–15 mg) as an analgesic.

Macaques received a preoperative and postoperative treatment regimen consisting of dexamethasone sodium phosphate (0.4 mg/kg) to reduce swelling and Cefazolin antibiotic (15 mg/kg) to prevent infection one day prior to and one week following the surgical date. Macaques received analgesic ketoprofen (10–15 mg) immediately following and two days after the procedure, as well as ibuprofen (100 mg) for 5 additional days.

#### Histology & immunochemistry

After a survival period of at least 4 weeks, the macaques were euthanized. They were first administered ketamine hydrochloride (10 mg/kg), followed by a lethal dose of sodium pentobarbital (100 mg/kg) intravenously. After euthanasia, the macaques were transcardially perfused with 1 L of physiological saline, followed by 3 L of 4% paraformaldehyde in 0.1 M phosphate-buffered saline. The brains were extracted and stored in 10% formalin while refrigerated. To prepare the brains for sectioning, they were cryoprotected in buffered formalin/glycerol and then sectioned into 40 μm coronal slices using a freezing microtome. Every fifth section was mounted on gelatin-coated slides, defatted, stained with thionine, and cover-slipped. ACC in the present study conforms with the regions identified as areas 24b, 24c, 32, and 25 of Carmichael and Price (Carmichael and Price 1994), with the exception that we did not examine cortex on the dorsal bank of the cingulate sulcus. These regions lie on the medial surface of the hemisphere and correspond approximately to cortex with borders defined as follows: the dorsal boundary was the fundus of the cingulate sulcus; the ventral boundary was ~ 2 mm dorsal to the fundus of the rostral sulcus; the rostral boundary was an imaginary line between the rostral tips of the cingulate and rostral sulci; and the caudal boundary was an imaginary line in the coronal plane passing through the caudal tip of the principal sulcus. A separate series was stained for acetylcholinesterase (AChE) (Geneser-Jensen and Blackstad 1971). Sections were then mounted on slides and cover-slipped.

### Data analysis

Brightfield images were acquired under a 40X objective using an Olympus VS 200 scanning microscope to examine the tissue. Cholinergic innervation of the ACC was assessed via the mean inverted pixel intensity of the AChE staining—referred to hereafter as ‘AChE staining’—quantified across interaural planes + 39 mm to + 24 mm in the intact and OFC lesion hemispheres in each macaque. The left and right hemispheres were matched in the anterior-posterior plane using anatomical landmarks. As a control, we assessed cholinergic AChE staining in the striatum. We included one to two samples per coronal section and sampled the ACC gyrus to avoid regions with curvature, i.e., gyral crowns and sulcal depths. Images were processed using ImageJ software (Schneider et al. 2012) and matched for background luminance using the calibration tool. The mean inverted pixel intensity was calculated for each coronal section from + 39 mm to + 24 mm. Further, we contrasted AChE staining between rostral ACC (+ 39 mm to + 32 mm) and caudal ACC (+ 31 mm to + 24 mm). Statistical analyses were performed to quantify the disparity in the AChE staining between the intact and lesioned hemispheres; significance was set at *p* < .05. Paired samples *t*-tests were performed on AChE staining between the hemispheres, as well as rostral and caudal portions of the ACC. To examine the laminar pattern of cholinergic projections to ACC, AChE staining was evaluated between the pial surface and white matter of each sampled region, and then averaged across all rostral-caudal levels in each hemisphere. To facilitate averaging, the cortical thickness of ACC sample regions was normalized across sections via interpolation to align the laminae across the anterior-posterior axis and AChE staining was plotted relative to the maximum for each macaque.

## Results

We performed unilateral aspiration removals of OFC areas 11, 13, and 14 in three macaques and compared cholinergic innervation of the ACC in the intact and OFC-lesioned hemispheres. The location and extent of the lesions were intended to replicate those described by (Baxter et al. 2000; Izquierdo et al. 2004); the caudal extent of the lesions was also consistent with our prior studies (Rudebeck et al. 2013; Izquierdo et al. 2004, 2005).

**Fig. 1:**
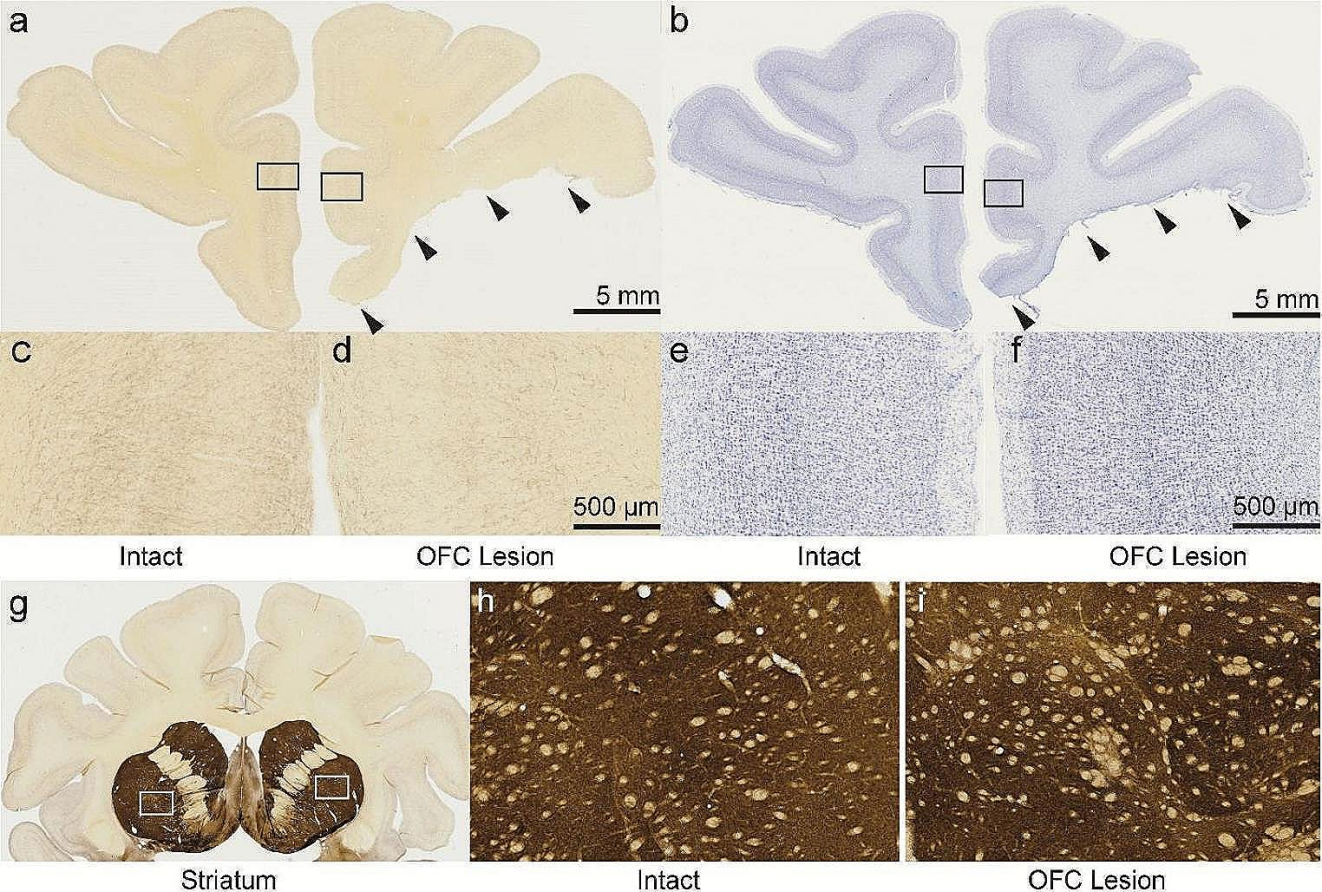
Immunohistochemistry of AChE (**a**) and thionine (**b**) stained brightfield images of a macaque brain. OFC was removed by aspiration lesion in the right hemisphere (black arrows) of monkey C. High magnification images of the ACC in the intact hemisphere (**c** & **e**) and OFC lesion hemisphere (**d** & **f**), corresponding to the black boxes in (**a**) and (**b**), respectively. AChE stained brightfield images of the striatum (**g**) in monkey C. High magnification images in the intact (**h**) versus OFC lesion (**i**) hemispheres, corresponding to the white boxes over the striatum in the left and right hemispheres in (**g**), respectively, were evaluated as a control. Left and right hemispheres were matched in the anterior-posterior plane prior to analysis

Cholinergic innervation was inferred via immunohistochemistry of AChE (Fig. 1a, c, d). Upon examination under a low power stereomicroscope, it was apparent that hemispheres with OFC removed exhibited lower levels of AChE staining in ACC compared to the intact hemispheres. There was no observable difference in the gross anatomy of the ACC between the two hemispheres, as visualized using a thionine stain (Fig. 1b, e, f).

**Fig. 2:**
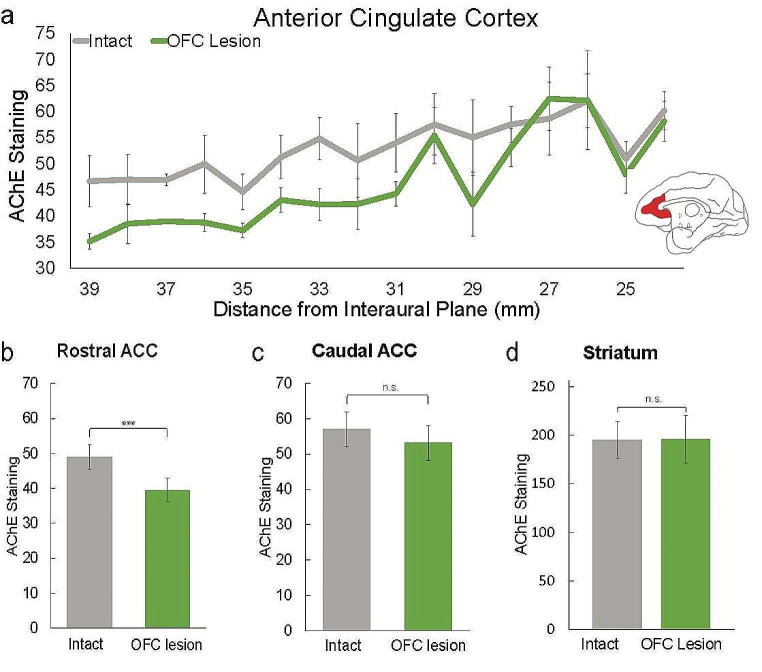
AChE staining (inverted mean pixel intensity) in (**a**) the ACC across coronal sections + 39 mm to + 24 mm anterior to the interaural plane in the intact hemisphere (grey) and the hemisphere with an OFC lesion (green). (**b**) Mean AChE staining in the rostral portion of the ACC, comprising + 39 mm to + 32 mm, and (**c**) in the caudal portion comprising + 31 mm to + 24 mm from the interaural plane. (**d**) AChE staining in the striatum. Data from 3 macaques. *** *p* < .01

To quantify these observations, we acquired an estimate of the AChE staining density in each macaque at 16 different anterior-posterior levels of the ACC. Consistent with our initial impression, the OFC lesioned hemisphere had significantly lower levels of AChE expression overall across all rostral-caudal levels of ACC (*F*(1, 28) = 15.35, *p* < .001) (Fig. 2a). AChE staining in the lesioned hemisphere was significantly lower than that in the intact hemisphere in the rostral portion of the ACC (*t*(7) = 13.40, *p* < .001), but not in the caudal portion (*t*(7) = 2.36, *p* = .08) (Fig. 2b, c). These effects were consistent across all subjects (Online resource 1). Cholinergic innervation of the striatum was evaluated as a control because this region receives most of its cholinergic inputs from local interneurons and, as such, was expected to remain unaffected by interruption of NBM projections. No hemispheric difference in AChE staining was observed within the striatum (*p* = .90) (Fig. 2d).

**Fig. 3:**
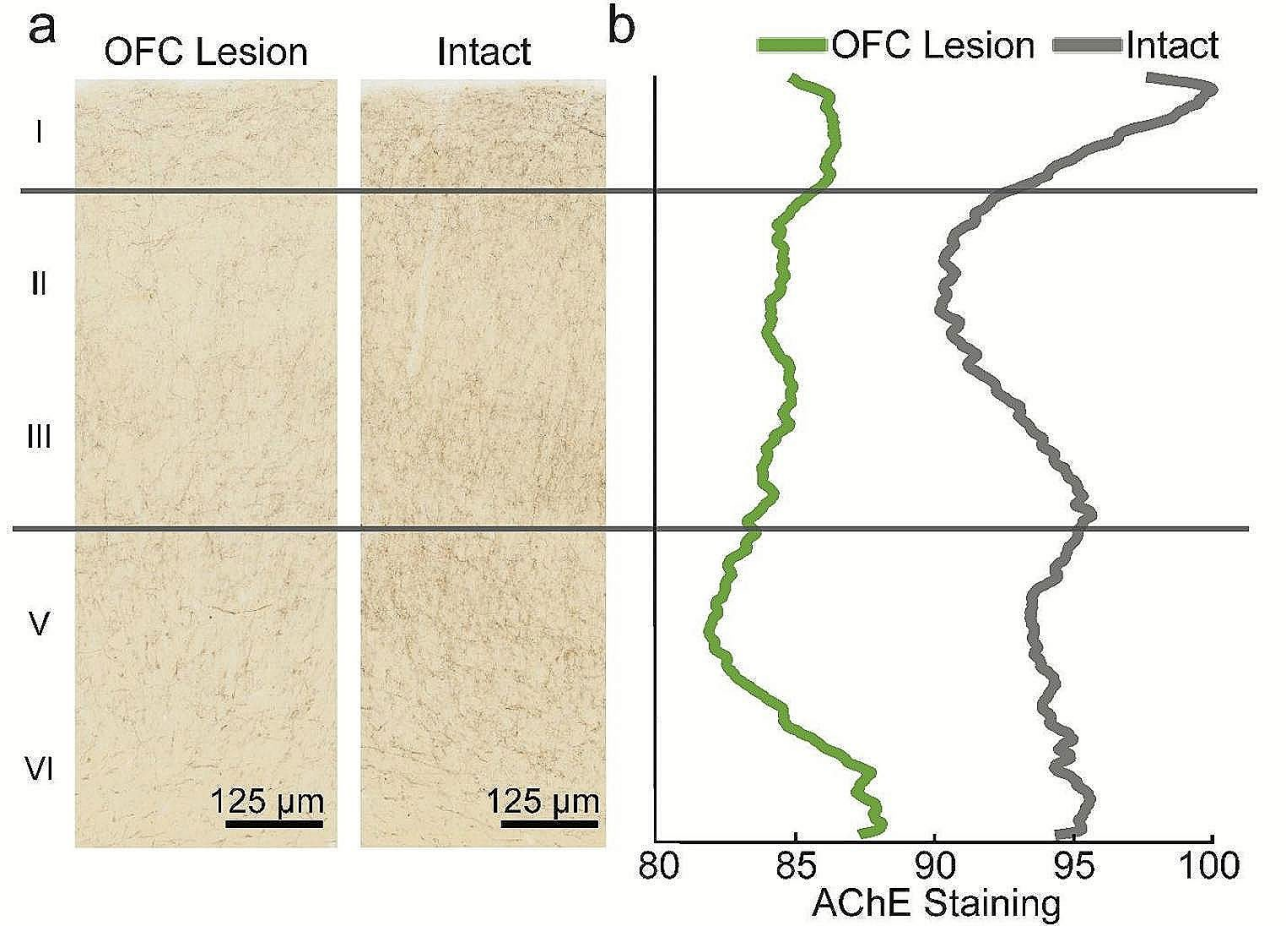
Laminar distribution of AChE staining in ACC. (**a**) Representative AChE stained brightfield images of the ACC in an OFC lesion hemisphere (left panel) and in an intact hemisphere (right panel). (**b**) AChE staining across cortical laminae averaged over all rostral-caudal levels of ACC in all macaques. AChE staining values represent relative mean inverted pixel intensity. Data from three macaques

Finally, changes in cholinergic innervation across laminae were examined. Consistent with prior reports (Carmichael and Price 1994; Preuss and Goldman-Rakic 1991), in intact hemispheres, the highest density of AChE staining was observed in layers I and III/V. Aspiration removal of OFC produced reduced AChE staining in all cortical layers; unsurprisingly, the greatest reductions were observed in layers I and III/V (Fig. 3). This effect was consistent across all subjects (Online resource 2).

## Discussion

The current study was designed to investigate mechanisms contributing to the disparities in behavioral outcomes between aspiration and excitotoxic lesions of the OFC in macaques, with a focus on the potential involvement of cholinergic fibers of passage. To gain a deeper understanding of how fibers of passage might be inadvertently compromised in our studies that make use of aspiration lesions, unilateral aspiration removals of OFC were performed, and cholinergic innervation in the ACC was compared between the lesioned and intact hemispheres. Additionally, we evaluated the local gradient of AChE staining/loss across the anterior-posterior extent of the ACC and across laminae. Aspiration removals of the OFC disrupted cholinergic staining in the ACC, consistent with the idea that these lesions disrupt fibers of passage.

The present findings demonstrate that cholinergic signaling is disrupted in frontal cortex regions outside of OFC consequent to OFC aspiration lesions. Thus, the findings are consistent with the idea that disruption of this projection could contribute to the significant impairments in cognitive flexibility and emotion regulation observed following aspiration lesions but not excitotoxic lesions of OFC (Rudebeck et al. 2013). Although it would have been ideal to study both cholinergic signaling and behavior (e.g., cognitive flexibility) in the same subjects, the experimental design precluded this approach. We reasoned that, given potential variability in staining between subjects due to individual differences in AChE expression and/or variability in the staining procedure, using an intact hemisphere as a within-subject control was essential to our analysis. Since unilateral lesions in macaques rarely result in long-term behavioral effects, we did not entertain acquiring behavioral data in the present study.

### Cognitive flexibility

Aspiration lesions of OFC impair object reversal learning, whereas excitotoxic lesions of the same region do not yield this effect (Rudebeck et al. 2013). Macaques with ACC ablation show impairments in object reversal learning (Chudasama et al. 2013), albeit milder than those that follow OFC aspiration lesions. The current findings are consistent with the hypothesis that interrupting the cholinergic fibers of passage in proximity to the OFC, as occurs during aspiration lesions, could be responsible at least in part for the effects of OFC aspiration removal on reversal learning. This possibility is consistent with the effects of selective cholinergic ablation in rats: the NBM sends cholinergic projections to regions throughout the prefrontal cortex, including to ACC; immunotoxic ablation of cholinergic neurons of the NBM via 192 IgG-saporin impairs reversal learning in rodents (Tait and Brown 2008). This evidence is consistent with the idea that acetylcholine could contribute to the regulation of cognitive and behavioral processes via modulation of ACC activity.

### Emotional regulation

Another task impaired by aspiration but not excitotoxic lesions of OFC is the approach-avoidance task. This task pits approach to retrieve food reward against withdrawal engendered by an innately aversive stimulus, a fake snake. Macaques with damage to the ACC sulcal cortex show altered responses on this task (Rudebeck et al. 2006). Thus, our findings regarding reduced cholinergic signaling in ACC consequent to aspiration lesions of OFC may contribute to the differential effects of OFC aspiration vs. excitotoxic lesions in this domain.

### Fibers of passage

The qualitatively similar behavioral impairments produced by lesions of the ACC and aspiration but not excitotoxic lesions of the OFC in both cognitive and affective domains, taken together with the present findings, are consistent with the interpretation that cholinergic projections to the ACC support at least some of the behaviors traditionally impaired by OFC ablation by aspiration, either alone or in combination with the loss of neurons in OFC.

Another possibility, not mutually exclusive with the first, is that the deficits in cognitive flexibility and emotion regulation observed after aspiration lesions of OFC result from a more complex pattern of inadvertent damage to fibers of passage; one involving VLPFC as well as ACC, and perhaps other frontal cortex areas associated with decision-making, motivation and emotion (Rolls 2023). Consistent with this idea, a recent study investigated the effects of OFC aspiration lesions of areas 11, 13, and 14 on gray matter volume in two macaques and found reductions in volume within a part of area 12/47 on the orbital surface (area 12o), as well as within ACC and parts of the dorsolateral prefrontal cortex (Sallet et al. 2020). Major fiber bundles coursing near the caudal OFC include the uncinate fascicle, external capsule, and cingulum bundle (Schmahmann and Pandya 2006; Jbabdi et al. 2013).

Not all behaviors that are supported by ACC are impaired by OFC damage; for example, ACC and OFC have dissociable roles in action-value and stimulus-value learning in both humans and macaques (Camille et al. 2011; Rudebeck et al. 2008). and the functions of ACC sulcal cortex and OFC have been doubly dissociated in tasks that assess multiple aspects of rule-guided behavior in monkeys (Buckley et al. 2009).

One limitation of our study is that, in some regions of the brain, AChE is not restricted to cholinergic neurons (Eckenstein and Sofroniew 1983). Although the presence of AChE is highly likely to reflect the local termination of cholinergic axons, and hence it is a reasonable proxy for cholinergic innervation (Calhoun et al. 2004), there is some evidence that AChE serves additional functions, such as degradation of Substance P (Chubb et al. 1980). Thus, we cannot rule out the possibility that AChE staining reflects a role other than neuromodulation of circuits in ACC. The present findings indicate that a fuller exploration of the white matter and neurotransmitter pathways affected by aspiration lesions of OFC, and direct comparison to effects following excitotoxic lesion of OFC, is warranted. Although OFC provides direct input to the NBM (Mesulam and Mufson 1984), we saw no evidence of a general downregulation of cholinergic projections. For example, AChE staining in caudal ACC was unaffected by the OFC lesion (Fig. 2b), supporting the conclusion that the reduction in cholinergic innervation to ACC was more likely driven by a severing of projections rather than an indirect effect on cell bodies.

Finally, our findings suggest a potential new interpretation regarding OFC damage in humans. Given the similar anatomical arrangement of white matter pathways coursing near OFC in humans and macaques (Jbabdi et al. 2013), our findings suggest that behavioral impairments observed in patients following OFC damage might be attributable, at least in part, to disruptions of fibers coursing nearby or through OFC en route to prefrontal cortex regions outside OFC. In theory, inadvertent disruption of fibers of passage could occur with any procedure or insult in the region of the caudal OFC: tumor removal, stroke, or ruptured aneurysms of anterior communicating arteries. If so, damage apparently limited to OFC could affect more widespread areas of prefrontal cortex, as appears to be the case in the present study with macaques.

## Conclusion

Unilateral aspiration lesions of OFC areas 11, 13 and 14 in macaques disrupted cholinergic fibers of passage and led to a decrease in AChE staining within the ACC, particularly in the more rostral portion of the ACC. The loss of AChE staining was disproportionately concentrated in layers I and III/V, layers that have the highest levels of endogenous AChE expression. These findings are consistent with the hypothesis that the impairments in cognitive flexibility and emotion regulation observed following aspiration lesions, but not excitotoxic lesions, of the OFC may be attributed in part to the interruption of cholinergic fibers of passage; however, further work is needed to confirm a causal relationship. The results have implications for findings in the clinic and demonstrate the translational utility of experimental research in macaques.

### Electronic supplementary material

Below is the link to the electronic supplementary material.

Supplementary Material 1

## Data Availability

The full dataset is available in the Figshare repository, DOI: 10.6084/m9.figshare.24499198.
