# Supplementary material for: Aspiration removal of orbitofrontal cortex disrupts cholinergic fibers of passage to anterior cingulate cortex in rhesus macaques
Source: Brain Struct Funct. 2024 Mar 19;229(4):1011–9. doi: 10.1007/s00429-024-02776-6 (PMC11003915; doi:10.1007/s00429-024-02776-6)
Supplement: Supplementary file 1 — Supplementary Material 1 [file 429_2024_2776_MOESM1_ESM.docx]

**Supplementary Information**

*Article Title*: Aspiration removal of orbitofrontal cortex disrupts cholinergic fibers of passage to anterior cingulate cortex in rhesus macaques

*Journal Name*: Brain Structure and Function

*Authors*: M. A. G. Eldridge, A. Mohanty, B. E. Hines, P. M. Kaskan, E. A. Murray

*Affiliation*: Laboratory of Neuropsychology, National Institute of Mental Health, National Institutes of Health, Bethesda, MD

*Corresponding author emails*: [mark.eldridge@nih.gov](mailto:mark.eldridge@nih.gov), [elisabeth.murray@nih.gov](mailto:elisabeth.murray@nih.gov)


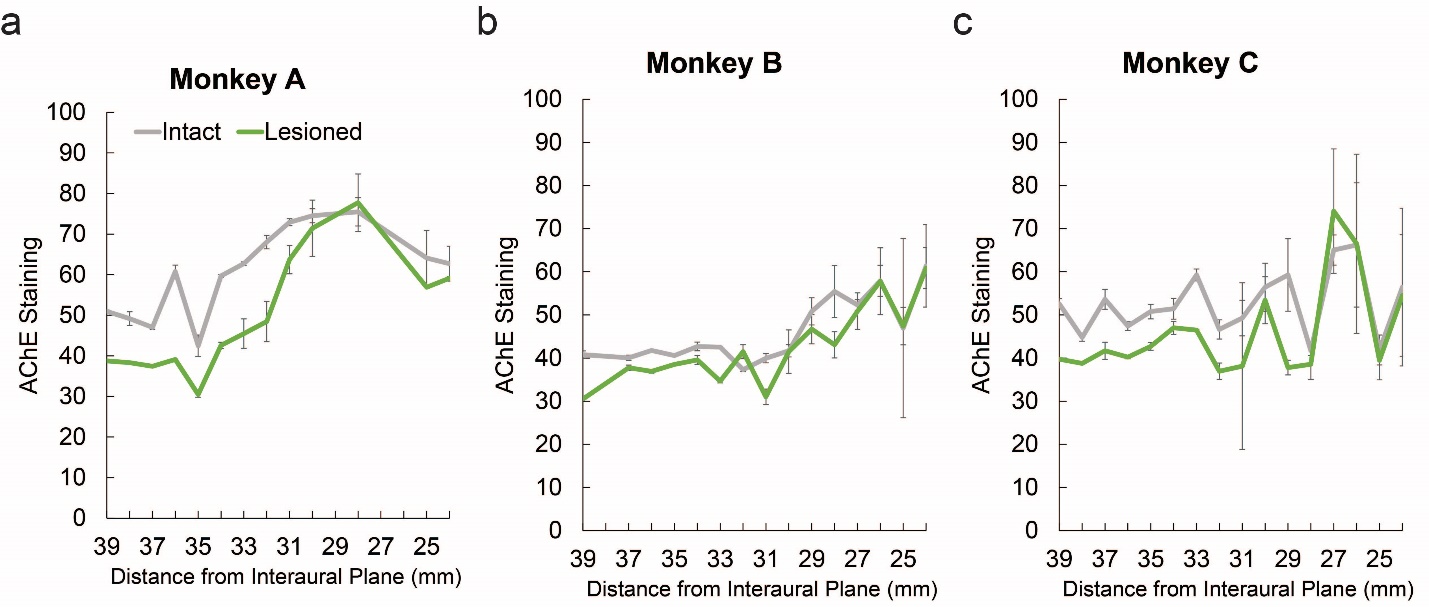


***Online Resource 1*** *AChE staining within ACC levels +39 mm to +24 mm in hemispheres with (grey) or without (green) OFC in (a) monkey A, (b) monkey B, and (c) monkey C. AChE staining values represent relative mean inverted pixel intensity.*


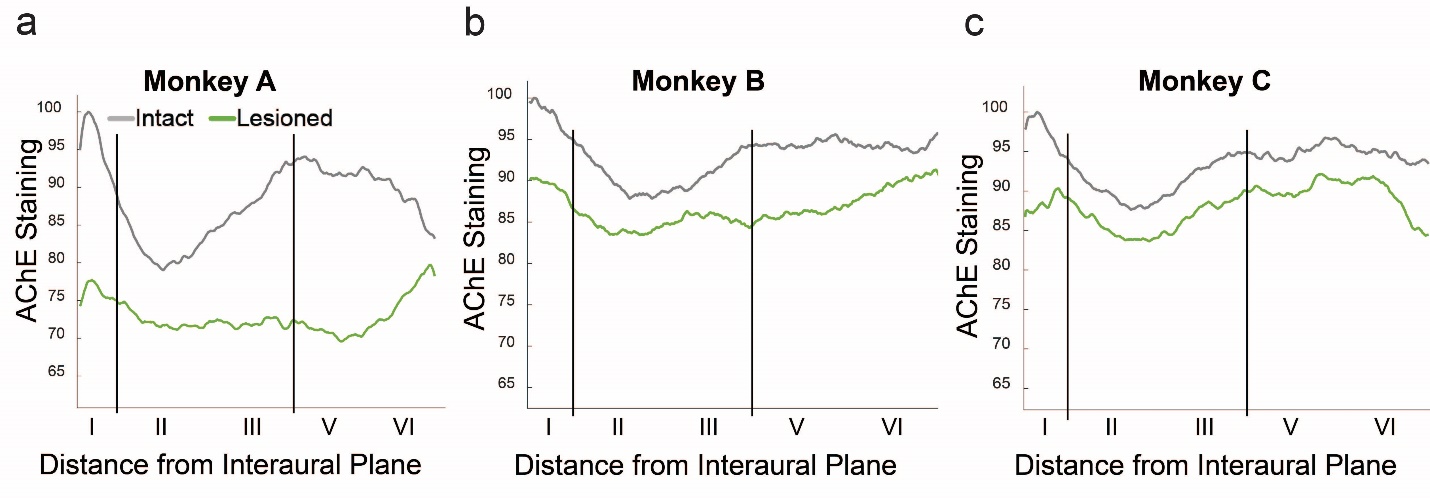


***Online Resource 2*** *Mean laminar distribution of AChE staining in the ACC in the hemisphere with (grey) and without (green) OFC, in (a) monkey A, (b) monkey B, and (c) monkey C. AChE staining values represent relative mean inverted pixel intensity.*
